# Factors facilitating the implementation of a clinical decision support system in primary care practices: a fuzzy set qualitative comparative analysis

**DOI:** 10.1186/s12913-023-10156-9

**Published:** 2023-10-26

**Authors:** Alexandra Piotrowski, Jana Coenen, Christian Rupietta, Jale Basten, Christiane Muth, Sara Söling, Viola Zimmer, Ute Karbach, Petra Kellermann-Mühlhoff, Juliane Köberlein-Neu, the AdAM study group, Marjan van den Akker, Till Beckmann, Benjamin Brandt, Robin Brünn, Kiran Chapidi, Truc Sophia Dinh, Lara Düvel, Benno Flaig, Mathias Flume, Ferdinand M. Gerlach, Paul Glasziou, Ana Isabel Gonzalez-Gonzalez, Daniel Grandt, Simone Grandt, Wolfgang Greiner, Reinhard Hammerschmidt, Sebastian Harder, Peter Ihle, Julia Jachmich, Renate Klaaßen-Mielke, Eva Leicher, Dorothea Lemke, Frank Meyer, Ingo Meyer, Beate S. Müller, Thomas Müller, Rafael Perera, Holger Pfaff, Johanna Richard, Bastian Surmann, Nina Timmesfeld, Hans J. Trampisch

**Affiliations:** 1 Center for Health Economics and Health Services Research, University of Wuppertal, Wuppertal, Germany; 2 Chair of General Practice II and Patient-Centeredness in Primary Care, Institute of General Practice and Primary Care, Faculty of Health, Witten/Herdecke University, Witten, Germany; 3 Jackstädt Center of Entrepreneurship and Innovation Research, University of Wuppertal, Wuppertal, Germany; 4 Queen’s Business School, Queen’s University Belfast, Belfast, UK; 5 Department of Medical Informatics, Biometry and Epidemiology, Ruhr University Bochum, Bochum, Germany; 6 Department of General Practice and Family Medicine, Medical School OWL, Bielefeld University, Bielefeld, Germany; 7 Institute for Medical Sociology, Health Services Research and Rehabilitation Science, Department of Rehabilitation and Special Education, Faculty of Human Sciences, University of Cologne, Cologne, Germany; 8 BARMER Statutory Health Insurance, Wuppertal, Germany

**Keywords:** Primary care, Implementation, Qualitative comparative analysis, Organizational behavior

## Abstract

**Background:**

Understanding how to implement innovations in primary care practices is key to improve primary health care. Aiming to contribute to this understanding, we investigate the implementation of a clinical decision support system (CDSS) as part of the innovation fund project AdAM (01NVF16006). Originating from complexity theory, the practice change and development model (PCD) proposes several interdependent factors that enable organizational-level change and thus accounts for the complex settings of primary care practices. Leveraging the PCD, we seek to answer the following research questions: Which combinations of internal and external factors based on the PCD contribute to successful implementation in primary care practices? Given these results, how can implementation in the primary care setting be improved?

**Methods:**

We analyzed the joint contributions of internal and external factors on implementation success using qualitative comparative analysis (QCA). QCA is a set-theoretic approach that allows to identify configurations of multiple factors that lead to one outcome (here: successful implementation of a CDSS in primary care practices). Using survey data, we conducted our analysis based on a sample of 224 primary care practices.

**Results:**

We identified two configurations of internal and external factors that likewise enable successful implementation. The first configuration enables implementation based on a combination of *Strong Inside Motivation*, *High Capability for Development*, and *Strong Outside Motivation*; the second configuration based on a combination of *Strong Inside Motivators*, *Many Options for Development* and the absence of *High Capability for Development*.

**Conclusion:**

In line with the PCD, our results demonstrate the importance of the combination of internal and external factors for implementation outcomes. Moreover, the two identified configurations show that different ways exist to achieve successful implementation in primary care practices.

**Trial registration:**

AdAM was registered on ClinicalTrials.gov (NCT03430336) on February 6, 2018.

**Supplementary Information:**

The online version contains supplementary material available at 10.1186/s12913-023-10156-9.

Contribution to the literature

- We provide empirical analyses on the relevance of internal and external factors for innovation implementation in primary care practices. Moreover, corroborating the PCD, our results demonstrate the need for a combination of internal and external factors to implement successfully.
- Furthermore, using QCA, we show that two different combinations of factors can likewise facilitate successful implementation. We therefore encourage future research to consider this equifinality.
- We demonstrate the applicability of both the PCD and QCA to study influencing factors in primary care settings.

## Background

Patient safety and preventable treatment errors are important issues in primary health care. Despite high treatment quality, vulnerable groups such as polypharmacy patients are still at risk of adverse events [1, 2]. Innovations such as clinical decision support systems (CDSSs) have the potential to mitigate this risk and, at the same time, reduce professionals’ workloads [3]. Understanding how those innovations can be implemented is therefore highly relevant for primary care research [1, 4, 5]. To implement an innovation, primary care practices must redesign workflows, redefine professional roles, and disseminate evidence-based knowledge. In other words, they need to change.

To understand change in primary care practices, previous research has employed a variety of approaches. Most of these approaches focus on either individual behavior [6], organizational characteristics [7–10], or (patient-related) performance [11–14] as enabling factors of change. Primary care practices are multidisciplinary and complex settings, however [4, 15–17]. Thus, to understand the change processes that are necessary to implement an innovation, an approach is needed that accounts for the multidimensionality of primary care practices.

This study contributes to the understanding of innovation implementation in primary care practices by leveraging the practice change and development model (PCD) [15, 18, 19] and qualitative comparative analysis (QCA) [20–24]. Originating from complexity theory, the PCD proposes several interdependent factors that enable organizational-level change and thus accounts for the complex settings of primary care practices [25, 26]. We used QCA to test how combinations of these interdependent factors enable innovation implementation. QCA is a configurational method that allows one to account for and shed light on the causally complex interrelations of factors [27, 28, 23]. By studying the implementation of a CDSS used for patients with polypharmacy, we seek to answer the following research questions: Which combinations of internal and external factors based on the PCD contribute to successful implementation in primary care practices? Given these results, how can implementation in the primary care setting be improved?

## Methods

### Theoretical background

Based on experience from research projects and adaptive systems theory, Miller and colleagues developed a theoretical model, the practice change and development model (PCD), to help them understand change in primary care practices [25, 26, 15].

**Fig. 1:**
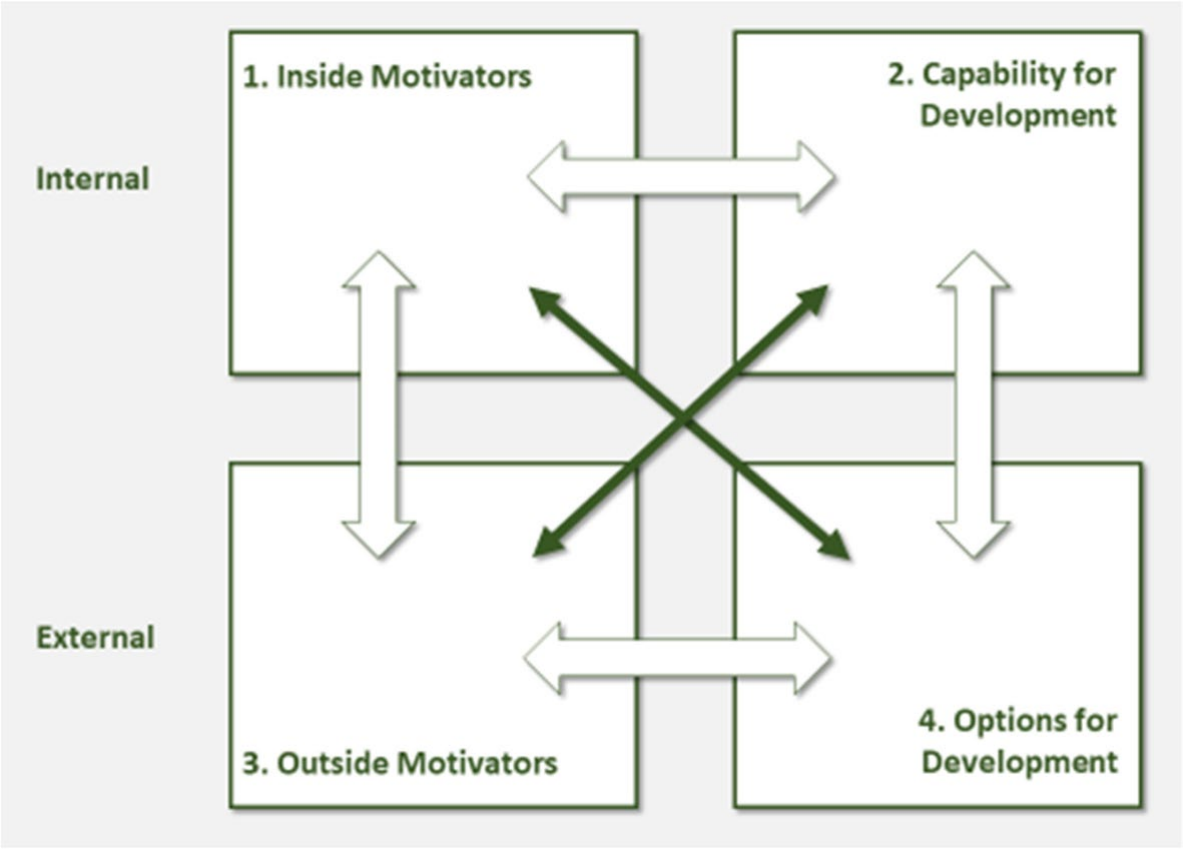
The practice change and development model (based on [25, 26], permission obtained from copyright holder)

Primary care practices are understood as complex, adaptive systems that are determined by four interdependent elements (Fig. 1). The upper two elements in Fig. 1 refer to the inside (inner setting) of a practice, the lower two to the outside (outer setting):

1. *Inside Motivators* are the practice’s own motivational drivers.
2. *Capability for Development* is the inner qualities that enable a practice to undergo change, including essential human resources and the adaptive reserve (or resilience) of the system, which is fostered by supportive leadership, a positive learning culture, sense making, communication, and good teamwork.
3. *Outside Motivators* are incentives for change or development that do not come from the practice itself but from external sources.
4. *Options for Development* are the perceived opportunities for change, for example a newly introduced intervention and its fit with existing needs.

The four PCD factors are interrelated [26]. External factors, for example an intervention (i.e., option for development), can trigger internal factors (i.e., motivation and capability for development). Internal factors, in turn, can impact external factors, for example by deliberately searching for outside support (i.e., outside motivators) or an opportunity to become active (i.e., options for development) [15, 29].

This ongoing exchange highlights the dynamic nature of change in primary care practices. Change in individual elements can affect all other elements as well as their contributions to the outcomes of the system. By uniting factors in a nonlinear way, the PCD is unique in the context of primary care.

### Study setting

This analysis was part of the AdAM project (German: Anwendung für ein digital gestütztes Arzneimitteltherapie- und Versorgungsmanagement, or “application of digitally supported drug-therapy and care management”), which was conducted between July 2017 and June 2021. In the AdAM project, the application of a new clinical decision support system (CDSS) in primary care practices was evaluated. The AdAM study design is described in detail elsewhere [30, 31]. In brief, the CDSS-based AdAM intervention addressed the medication management of multimorbid patients with polypharmacy performed by physicians (general practitioners). Participating physicians performed at least one medication review using a clinical decision support system (CDSS) with access to claims data from one German statutory health insurance company (BARMER). The CDSS was implemented in 676 participating primary care practices in Germany (Westphalia-Lippe region). General practitioners (with or without specialization) from the study region with at least 10 eligible patients and fulfilled contractual obligations were eligible to participate in the study (N = 925) [30]. The primary objective of the project was to decrease hospitalization and mortality rates among polypharmacy patients compared to routine care. The primary objective was investigated with a stepped-wedge cluster randomized controlled trial (SW-CRT). The present study deals with the implementation of the CDSS and its feasibility (Additional file 1).

The project was funded by the Innovation Fund of the German Federal Joint Committee (01NVF16006). AdAM was approved by the Ethics Commission of the Medical Association North Rhine (approval date July 26, 2017; approval no. 2017184) and registered on ClinicalTrials.gov (NCT03430336) on February 6, 2018 (https://clinicaltrials.gov/ct2/show/NCT03430336). Written informed consent was obtained from all participants (or their parent or legal guardian in the case of children under 16). All methods were performed in accordance with the relevant guidelines and regulations.

### Data collection

Data were collected in a cross-sectional postal survey from September to December 2020. The survey was designed for the purpose of this study (see Additional file 2). It contains self-designed items as well as validated measures. For data protection, questionnaires were distributed by the Westfalen Lippe association of health insurance physicians (KVWL) to physicians actively participating in the project. At that time, the SW-CRT was completed, and all practices had reached intervention status. Following Dillmann’s approach [32], KVWL sent two written reminders after two and four weeks. The physicians rated the items on a 5-point Likert scale (1 = *strongly disagree*, 3 = *neither agree nor disagree*, 5 = *strongly agree*).

For descriptive analysis, we utilized three items from the main study as secondary data aggregated at the practice level (1: Share of participating patients with medication changes per practice, 2: Median number of medication warnings per patient prior to intervention, 3: Median number of medication warnings after intervention; see also Table 5 in the “Results” section).

### Response rate and case selection

After data cleaning and aggregation to the practice level in the case of joint practices, we included 224 cases (practices) in the present analysis (response rate: 44.53%).

Table 1 summarizes the characteristics of the included practices in comparison to practices participating in the overall AdAM study.

**Table 1:** Characteristics of AdAM participants and practices included in the QCA

	**AdAM practices**	**Practices included in QCA**
**Participants (*n*)**	676	224
**Age of physicians (mean [SD])**	55.11 (7.71)	53.58 (8.48)
**Gender (*n* [%])**		
Female only	163 (24.11%)	48 (21.43%)
Male only	404 (59.76%)	128 (57.14%)
Mixed gender	109 (16.12%)	48 (21.43%)
**Years of work experience in this organization (mean [SD])**	19.0 (9.14)	18.22 (8.67)
**Form of cooperation (*n* [%])**		
Joint practice	227 (33.58%)	93 (41.52%)
Solo practice	444 (65.68%)	129 (57.59%)
Medical Care Center	5 (0.74%)	2 (0.09%)
**Further physicians per practice (mean [SD])**	1.79 (1.11)	1.83 (1.10)

“Participants” denotes the number of participating practices; “age” denotes the mean of the average for each practice’s physicians; “years of work experience in this organization” describes the mean of the average for each practice’s physicians

Table 1 shows that, in regard to the selected characteristics, the survey respondents did not significantly differ from all AdAM participants. They were therefore considered to be representative of all AdAM participants, allowing us to generalize our results to all AdAM participants.

### Fuzzy-set qualitative comparative analysis

To answer the research questions, we applied fuzzy-set QCA using the QCA package in R [33]. QCA uses a minimization algorithm that builds on Boolean algebra to identify configurations of conditions that are sufficient for a previously defined outcome [27].

QCA uses its own terminology: for example, “conditions,” a term that is analogous to the term “independent variables” in a correlational model; and “outcome,” a term that is analogous to the term “dependent variable.”

The set-theoretic origin of QCA, which distinguishes it from correlational approaches, gives it two analytical advantages for our research aim. First, QCA features conjunctural causation, allowing one to assess the impact of multiple conditions combined [27]. This feature fits the theoretical assumptions of the PCD: While the presence of any condition is expected to contribute to the presence of implementation success, the (initial) absence of one condition not necessarily contributes to the absence of implementation success. For example, if a primary care practice is (initially) not motivated to change, an external option for development may give the impetus for change instead and thus compensate the lack of motivation.

Second, QCA features equifinality, enabling to identify multiple configurations of conditions that are associated with the outcome [27]. This is important because we assume to identify more than one configuration that allows practices to implement successfully. This feature is in line with the PCD, which proposes that interrelated conditions contribute to implementation success.

### Measures

We operationalized the four PCD elements “Inside Motivators,” “Capability for Development,” “Outside Motivators,” and “Options for Development” with indicators from our standardized questionnaire (see Table 2). For single-item variables, we calculated the arithmetic mean. For the validated instruments (ORIC [34, 35] and PAR [18]), we included the respective score.

**Table 2:** Survey items used for fsQCA model

Condition	Measures
1. Strong Inside Motivators	German version of the Organizational Readiness for Implementing Change (ORIC) scale [34, 35]
2. High Capability for Development	Practice Adaptive Reserve (PAR) measure [18]^a^ additional self-designed items: • We have the time resources to adequately dedicate ourselves to such a project • We have the human resources (education and skills of the employees) to adequately dedicate ourselves to such a project
3. Strong Outside Motivators	Self-designed items: • The communication about the project by the project management (KVWL and BARMER) motivated me to introduce AdAM into my primary care practice • The attempts by the project management to contact me during the project motivated me to use AdAM
4. Many Options for Development	Self-designed items: • The AdAM software is an enhancement of our existing technological equipment • My expectations regarding the use of the AdAM software have been fulfilled • The AdAM software gives me confidence in my decisions and actions in the context of my patients’ drug therapy
Outcome	Self-designed item: • I used the AdAM software with all enrolled patients whenever necessary from my perspective

^a^The PAR was translated from English to German for the first time. Psychometric properties were tested for the current study; a full validation is still pending

The outcome is successful implementation, which is assessed using the single-item measure “I used the AdAM software with all enrolled patients whenever necessary from my perspective” on a 5-point Likert scale (1 = *strongly disagree*, 3 = *neither agree nor disagree*, 5 = *strongly agree*). The wording ensures that the physicians actually used the intervention with enrolled patients. In addition, the expression “whenever necessary from my perspective” implies that the use was perceived to be appropriate.

### Calibration

Due to its set-theoretic foundation, QCA requires the transformation of measures into sets. Thus, we assigned a score between 0 (non-membership) and 1 (full membership) to every expression of our variables. We used the direct method of calibration to transform the raw data into set-membership scores [36, 37]. This method uses a logistic function and requires the specification of three anchor points: a fully-in point, which translates into a set-membership score close to 1; a fully-out point, which translates into a set-membership score close to 0; and a point of maximum ambiguity, which translates into a set-membership score of 0.5 [38].

As described above, we assessed all items using 5-point Likert scales. Given this scale, we set the following calibration anchors (for the conditions and the outcome). Using an exclusion cutoff of 2, we assigned a set-membership score close to 0 to every practice that (strongly) disagreed with the respective item. Using an inclusion cutoff of 5, we assigned a set-membership score close to 1 to every practice that strongly agreed with the respective item. With a crossover point of 3.5, we assigned a set-membership score greater than 0.5 to every practice that agreed with rather than was unsure about the respective construct.

### Truth table

The following presentation of results was guided by the standards of good practice by Schneider and Wagemann [36] (Additional file 3) and the STROBE Checklist [39] (Additional file 4). First, QCA compiles all logically possible condition combinations in the truth table (Table 3). Each condition can be present or absent, resulting in 16 logically possible combinations (represented by 16 rows). After we assigned each case to one row, every row was represented by at least one case. Given this fully populated truth table, our results do not face the problem of limited diversity.

**Table 3:** Truth table AdAM fsQCA

	**Strong Inside Motivation**	**High Capability for Development**	**Strong Outside Motivation**	**Many Options for Development**	**Output value**	***n***	**incl**	**PRI**
1	0	0	0	0	0	72	0.573	0.321
2	1	0	0	0	0	12	0.766	0.451
3	0	1	0	0	0	15	0.745	0.409
4	1	1	0	0	0	4	0.820	0.510
5	0	0	1	0	0	13	0.825	0.508
6	1	0	1	0	0	9	0.859	0.591
7	0	1	1	0	0	6	0.873	0.590
8	1	1	1	0	1	4	0.882	0.646
9	0	0	0	1	0	6	0.816	0.499
10	1	0	0	1	1	8	0.870	0.642
11	0	1	0	1	0	2	0.867	0.560
12	1	1	0	1	0	11	0.863	0.629
13	0	0	1	1	0	12	0.869	0.612
14	1	0	1	1	1	11	0.895	0.703
15	0	1	1	1	0	6	0.877	0.609
16	1	1	1	1	1	33	0.883	0.754

*n number of cases in configuration, incl sufficiency inclusion score, PRI proportional reduction in inconsistency*

Second, using Boolean algebra, QCA minimizes the combinations shown in the truth table to a parsimonious solution term. For inclusion in this minimization process, we set a frequency, a raw consistency, and a proportional reduction in inconsistency (PRI) threshold. The frequency threshold was set to 1. In line with the standards of good practice in QCA, we set the raw consistency threshold to 0.8 [40]. To ensure that a configuration contributes to the presence of the outcome rather than its absence, we additionally set the PRI threshold^1^ to 0.64 [37, 33].

## Results

### Configurations sufficient for the outcome

The Boolean minimization^2^ resulted in two equifinal configurations sufficient for the outcome (Table 4). The black filled circles indicate present conditions; the crossed-out circle indicates an absent condition. Empty cells indicate conditions that were not relevant for the respective solution and could be either present or absent. The parameters of fit indicate a high overall model quality. The overall solution consistency of 0.85 indicates a consistent link of both solutions to the outcome, given the common practice of accepting all consistency scores ≥ 0.80 [41]. Additionally, the coverage score of 0.54 shows that the solution has high empirical relevance.

**Table 4:**
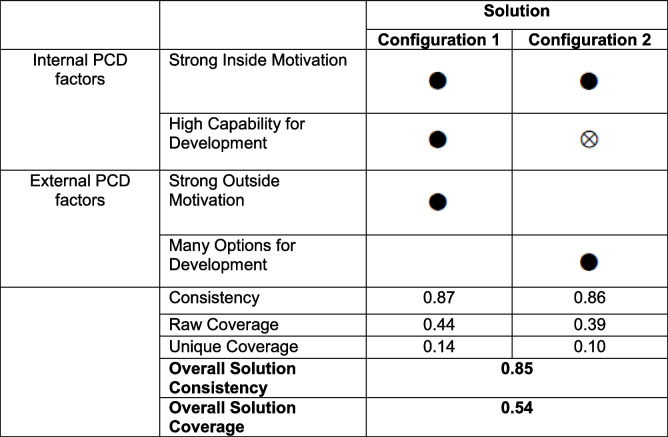
Results of the AdAM fsQCA

Table adapted from Fiss 2011 [38]

Configuration 1 shows that practices can implement innovation successfully due to a combination of *Strong Inside Motivation*, *High Capability for Development*, and *Strong Outside Motivation*. We called this solution *“Capability meets motivation.”*

Configuration 2 shows that, in the absence of *High Capability for Development,* practices can still implement innovation successfully due to a combination of *Strong Inside Motivators* and *Many Options for Development*. We called this solution “*Overcoming lack for capability for change.”*

We tested the robustness of our results according to the Robustness Test Protocol by Oana and Schneider (see Additional file 6) [42].

### Additional case knowledge

To understand how successful implementation was possible in the context of the AdAM project, we took a deeper look on the underlying mechanisms of our two equifinal configurations of conditions *Capability meets motivation* and *Overcoming lack for capability for change*. We did so by examining one ideal case for each identified configuration. Ideal cases are single cases that best correspond to the respective identified configuration and outcome [37].

To interpret our identified configurations, we conducted additional descriptive analyses of the organizational and structural characteristics of the practices. Table 5 lists the results for both ideal cases.

**Table 5:** Organizational and structural characteristics of ideal cases

Item	Ideal case of configuration 1: SinglePractice	Ideal case of configuration 2: JointPractice	Explanation
Physicians per practice	1	3	
Number of employees	5	9	
Form of cooperation	2	1	1 = joint practice, 2 = single practice
Participation in AdAM training	no	yes	
Percentage of staff involved	100%	11%	In Case 1, 5 staff members were involved in the use of the intervention; in Case 2, only 1 was
Share of participating patients with medication changes per practice	73%	33%	
Median number of medication warnings per patient prior to intervention	3.49	5.33	
Median number of medication warnings after intervention	2.98	5	

*Note: Configuration Types 1 and 2 are group variables. Values are displayed as average or median*

The ideal case for “*Capability meets motivation*” (Configuration 1) is a single practice called “SinglePractice” with five employees. The entire team of SinglePractice, consisting of five employees, was involved in the project, representing 100% participation. However, it is noteworthy that no one from SinglePractice attended the AdAM training.

The physician was responsible for the implementation of AdAM and was in charge of the team. As a result, SinglePractice successfully changed the medication of 73% of its enrolled patients and reduced the median medication warnings per patient from 3.49 to 2.98.

The ideal case for *“Overcoming lack for capability for change”* (Configuration 2) is a joint practice with nine employees, including three physicians. We refer to it as “JointPractice.” In JointPractice, one employee (11%), who is a physician, participated in the AdAM project and underwent training before introducing the software to the practice. Unlike SinglePractice, the entire team of JointPractice did not participate in the project.

Despite the limited participation, JointPractice still managed to change the medication of 33% of its enrolled patients. Additionally, similar to SinglePractice, JointPractice achieved a reduction in the median number of risk reports per patient, which decreased from 5.33 to 5.

## Discussion

To gain insight into the factors that facilitate the successful implementation of a CDSS-based intervention in primary care practices, we identified two specific configurations of conditions that contribute to implementation success. These findings emphasize the importance of considering the combined effects of multiple conditions rather than focusing on individual factors alone. It supports the fundamental premise of the PCD that these factors are interconnected and that strengthening individual factors has a positive impact on the entire system [26, 15]. In order to gain a deeper understanding of how these identified configurations enable implementation success, we analyze the ideal cases and develop theoretical explanations for the underlying patterns in each configuration.

### Configurational patterns for successful implementation

Capability meets motivation demonstrates that practices capable of changing (i.e., *High Capability for Development*) and with strong internal and external motivation (i.e., *Strong Inside Motivators* and *Strong Outside Motivators*) can successfully implement an innovation. Our ideal case, SinglePractice, exemplifies how the interaction of these conditions facilitates implementation success.

*High Capability for Development*, which is a component of Configuration 1, encompasses elements such as teamwork, communication, an effective learning culture, and sense-making [26, 18]. These aspects are reflected in SinglePractice, as indicated by its set membership and the involvement of the entire team. By making AdAM a collective task, SinglePractice was able to effectively treat a larger proportion of patients.

The findings indicate that involving the entire team is a viable strategy for achieving successful outcomes. Many intervention designs primarily concentrate on physicians, neglecting the broader team. Future projects could enhance their effectiveness by placing greater emphasis on the team and its individual members, considering factors such as the specific roles and functions of team members in the implementation process. Additionally, it is important for projects to take into account the unique organizational context in which they are implemented [15, 16, 19]. By considering the contextual factors, tailored and context-specific implementation strategies can be developed [43].

The absence of relevance for *Many Options for Development* in SinglePractice suggests that the presence of *Strong Inside Motivation, Strong Outside Motivation*, and *High Capability for Development* in itself provided the necessary impetus and resources for successful implementation. This indicates a certain degree of independence from specific project specifications or external options for development. By discussing SinglePractice as an ideal case, we can gain insights into how the configuration of motivational factors and the capability for change collectively contribute to enabling successful implementation.

Overcoming lack for capability for change shows that primary care practices can successfully implement an innovation even if they face less-than-ideal internal conditions (i.e., the absence of *High Capability for Development*). The absence of *High Capability for Development* indicates that the practices did not have enough time or staff for the project and were also lacking adaptive reserve. This is supported by JointPractice, the ideal case for the configuration *Overcoming lack for capability for change*, which did not involve the entire team in the project (see “Additional case knowledge” section) and – possibly therefore – treated fewer patients than SinglePractice.

Carl May describes implementation as a “negotiation of context” [44], wherein certain implementation activities must occur. It appears that these activities are more complex and time-consuming in larger teams compared to single practices. To achieve success in the face of a lack of capability, practices must perceive the innovation as useful (i.e., *Many Options for Development*) and demonstrate a willingness to change (i.e., *Strong Inside Motivation*). JointPractice best represents how the interplay of these conditions enables implementation success despite the absence of high capability levels. Unlike SinglePractice, the strong internal motivation appears to have driven JointPractice to attend the AdAM training. This training may, in turn, have strengthened the perceived potential of the intervention (*Many Options for Development*). As a result, the combination of their internal motivation and the heightened perception of potential contributed to the successful implementation of AdAM in JointPractice.

### Adding up to existing literature on CDSS implementation

There is a large body of literature that explores barriers and facilitators to CDSS implementation. This literature provides valuable insights into implementation activities, but also points to research needs that we have sought to address.

Damoiseaux-Volman and colleagues [45] conducted a systematic review of CDSS implementation within the inpatient sector. Data extraction was conducted based on the Grol and Wensing Implementation of Change Model [46]. One important conclusion was that interventions employing multiple implementation strategies yielded better outcomes compared to studies with a single-faceted approach. This aligns with our findings that not only one configuration of organizational properties contributed to achieving the desired implementation outcome [45].

Another review [47] that described barriers and facilitators to CDSS implementation in hospital settings utilized the 'Nonadoption, Abandonment, Scale-up, Spread, and Sustainability' (NASSS) framework [48]. The NASSS Framework encompasses seven implementation deterministic domains. Organization-related barriers and facilitators were reported in 27% of the examined studies, underscoring their relevance for effective implementation. At the same time, this highlights an underreporting in the remaining studies [47]. The results of this review emphasize the importance of organizational readiness for change and capabilities for innovation, consistent with our own findings: Readiness for change was present in both reported configurations. Capabilities for Development also played a role in both configurations: They were present in our Configuration *Capability meets motivation* but absent in our configuration *Overcoming lack of capability for change*. Interestingly, none of the examined studies reported on organizational resilience [47], despite our results indicating its high potential significance. This reveals a significant research gap.

However, it is important to note that the NASSS Framework covers a broader range of domains compared to the PCD. Nonetheless, it does not provide information about the interplay between these domains and subdomains regarding primary care practices.

A review by Westerbeek and colleagues [49] provides valuable insights into facilitating and hindering factors related to the acceptance of information systems, such as CDSS, in primary care. For data extraction, the 'Human, Organization, and Technology-fit' (HOT-fit) model [50] was applied. The results indicate that perceived usefulness is a crucial factor for the acceptance and successful use of CDSS, supporting the findings of our research as our outcome had a focus on sense making. However, the HOT-fit model does not encompass external factors, leaving an important perspective missing. Our results demonstrate that implementation processes involve a dynamic interplay of external and internal factors. Furthermore, the HOT-fit model primarily emphasizes technical aspects and overlooks human factors such as readiness and resilience. Nevertheless, our analysis demonstrates the high relevance of these factors in the context of successful CDSS implementation.

### Practical implications

To derive ways to improve implementation in primary care practices, we draw on the similarities of our identified configurations. First, in both configurations, we see the readiness to change (i.e., *Strong Inside Motivators*) as beneficial for successful implementation [51]. The occurrence across configurations highlights the importance of practices being change-ready. Our findings thus corroborate the literature, which states that change-ready organizations are more likely to initiate change, implement it with greater commitment, and invest more effort [51]. We recommend that future projects focus more strongly on this aspect, for example through a readiness assessment. Further research can help to understand how readiness to change can be strengthened before starting a project [52].

A second similarity of the two configurations is the combination of internal and external factors. While the two configurations feature different conditions, they both require (at least) the presence of one internal and one external condition. Thus, our findings imply that primary care practices need both internal and external support for successful implementation. Thus, as well as inside motivation, our findings suggest that future implementation projects should always ensure additional external enabling factors. Our findings confirm that practices not only react to input from the outside, but also create their own learning environment [15]. These internal efforts should be acknowledged and adopted by project leaders and evaluators. For example, future implementation projects may raise awareness of change processes and motivate and involve the staff through specifically assigned roles and tasks. Moreover, regular exchange (such as feedback loops) should become an integral part of implementation projects.

### Methodological implications

Several alternative models and frameworks could have been considered for our analysis. In the following, we examine some possible alternatives: The Behavior Change Wheel by Susan Michie and colleagues [53], explains translational activities, such as the implementation of innovation, through behavior change at an individual level, utilizing three main determinants: Capability, motivation, and opportunity. Interestingly, these determinants are also mirrored in the PCD (inside motivators, capacity for development, and options for development), but they are operationalized within the context of both the inner and outer settings of primary care practices. This elevates the PCD to an organizational level, which we regard as a significant strength of the PCD.

Our second example is the Grol and Wensing Implementation of Change Model [46], which offers practitioners and researchers a comprehensive guide with step-by-step actions for implementing change. A significant advantage of this model is its incorporation of various feedback loops. However, its stepwise presentation of actions may render it less suitable for explaining dynamic, non-linear settings.

The Consolidated Framework for Implementation Research (CFIR) [54, 55] serves as our third example. It also derives from implementation science and is a commonly used model. The primary strength of the CFIR is its integration of the inner and outer settings, as well as, in the newest version, implementation and innovation factors [55].

Besides this wide range of contextual factors, the CFIR also takes individual factors into account, namely Capability, Opportunity, and Motivation (which we also found in the PCD and the behaviour change wheel). In general, we consider the CFIR to be a very suitable framework for implementation research.

However, for our analyses, we chose to work with the primary care-associated PCD in order to more precisely focus on the implementation factors specific to the setting and to acknowledge the substantial potential of incorporating complexity-based models into primary care research, as emphasized in existing literature [15–17, 19, 44, 56]. Simultaneously to highligthing the potential of complexity science, there is also a call for non-linear evaluation approaches that align with these characteristics [17, 20, 21, 57, 58].

We aim to answer this call by bringing together a complexity theory-based model (PCD) with a non-linear method (QCA). The case-based perspective and configurational understanding of QCA enabled us to identify two equifinal configurations of conditions, highlighting the fundamental notion of the PCD that primary care practices are dynamic and complex settings, where change, such as implementation efforts, does not follow a linear path [26, 15].

By identifying complex causal patterns with QCA, we demonstrated that the PCD effectively explains changes in a primary care setting. Our findings support the basic assumption of the PCD that practices are adaptive learning systems that evolve through an interaction of internal and external factors [26, 15].

Furthermore, by combining QCA and PCD we believe we have been able to address some existing research gaps such as the mechanisms underpinning the interplay of organizational factors and the relevance of organizational readiness to change and organizational resilience in particular.

QCA is capable of identifying equifinal patterns in a dataset and attributing context-sensitive causality, going beyond the single-cause attribution that statistical methods would typically provide [37, 59]. Statistical methods are often preferred because of their high level of external validity and generalizability. Our results, as demonstrated in the results section, are consistent and robust, highlighting the generalizability of our findings. In a comparison between QCA and logistical regression, Befani concluded that while QCA demonstrated equal strength in external validity, it also provided a deeper understanding of the mechanisms by which outcomes occurred and better explained the complexity of causal relations [59]. In summary, for our analyses, QCA combined the analytical advantages of both qualitative and quantitative approaches.

It is worth noting that for researchers looking to delve deeper into qualitative methodologies beyond QCA, there exists a range of methodological alternatives, including Realist Evaluation [60, 61], Contribution Analysis [62] or Process Tracing [63, 64].

### Strengths and limitations

The main strength of our study is our methodological approach, demonstrating a beneficial correspondance between our theoretical model (PCD) and our empirical method (QCA). Our study allowed us to identify PCD key components that led to successful implementation in primary care practices and shed light on the underlying mechanisms for successful implementation.

Our results are consistent and robust (see “Results” section and Additional file 4). Studies with a similar focus – for example, by Hill [65], Yakovchenko [66], and Ziemann [22] – and a systematic review on the use of QCA in public health research [24] support the potential of QCA in analyzing complex causal conditions for evaluating healthcare programs.

Despite offering valuable insights, our study also has its limitations. First, the analysis lacks a time-related component, as the data are based on a cross-sectional survey. Time is an important factor in understanding development processes in a complex system [16] and is also reflected in the PCD [25, 26]. Thus, we encourage future researchers to complement our results by investigating, for example, the longevity of changes enabled by both our identified configurations. Second, the PCD was a valuable model for our study. However, there is room for interpretation in its operationalization. Different measures might have yielded different results. While we selected the PCD due to its primary care focus, other theoretical models or frameworks would have been suitable as well (e.g. The CFIR [54, 55], or The NASSS Framework [48] for a broader perspective, or the HOT-fit model [50] for a more technological / oranizational perspective). We encourage future studies to contribute to our research by applying a different theoretical basis. Third, our outcome measure covers a specific part of the facets that successful implementation might include. While we focused on sense making, other operationalizations of implementation success are possible (see the suggestions of Proctor and colleagues [67]). Thus, we recommend that future research apply our approach to other outcome measures to validate our results.

## Conclusion

In line with the PCD, our results demonstrate the importance of the interplay of internal and external factors for implementation outcomes. We identified two types of configurations, *Capability meets motivation* and *Overcoming lack for capability for change*, which reveal different ways to achieve a desired outcome.

The innovative potential of primary care practices has received comparatively little attention in the literature. The PCD provides a comprehensive framework to explore the change-enabling factors in primary care practices.

Moreover, QCA allows the identification of configurations of factors associated with successful change. By applying both the PCD and QCA, we contributed to an understanding of the causally complex interactions of change-enabling factors in the primary care setting. In so doing, we exemplified the benefit of applying both the PCD and QCA to the study of primary care practices.

### Supplementary Information

**Additional file 1.** Sub study: Qualitative Comparative Analysis (QCA).

**Additional file 2.** AdAM survey (translated version).

**Additional file 3.** Checklist based on STANDARDS OF GOOD PRACTICE IN QUALITATIVE COMPARATIVE ANALYSIS (QCA) AND FUZZY-SETS by Schneider & Wagemann 2007 (doi:10.1163/156913210X12493538729793).

**Additional file 4.** STROBE Statement—Checklist of items that should be included in reports of observational studies.

**Additional file 5.** Necessity analysis.

**Additional file 6.** Robustness test.

## Data Availability

Data are available from the corresponding author upon reasonable request.

## Abbreviations

AdAM: Application of digitally supported drug-therapy and care management (German: *Anwendung für ein digital gestütztes Arzneimitteltherapie- und Versorgungsmanagement*)
CDSS: Clinical decision support system
fsQCA: Fuzzy set qualitative comparative analysis
ORIC: Organizational Readiness for Implementing Change
PAR: Practice Adaptive Reserve
PCD: Practice change and development model
SW-CRT: Stepped-wedge cluster randomized trial
QCA: Qualitative comparative analysis
KVWL: Westfalen Lippe association of health insurance physicians (German: *Kassenärztliche Vereinigung Westfalen-Lippe*)

## Footnotes

1 The PRI threshold was chosen from the truth table. Two rows of the truth table are located between .64 and .65.

2 Before analyzing the truth table, we conducted an analysis of necessity. No condition (or its negation) is by itself necessary for the presence of the outcome (Additional file 5). Thus, we continued with our configurational analysis.
