# Supplementary material for: Factors facilitating the implementation of a clinical decision support system in primary care practices: a fuzzy set qualitative comparative analysis
Source: BMC Health Serv Res. 2023 Oct 26;23:1161. doi: 10.1186/s12913-023-10156-9 (PMC10605331; doi:10.1186/s12913-023-10156-9)
Supplement: Supplementary file 1 — Additional file 1. Sub study: Qualitative Comparative Analysis (QCA). [file 12913_2023_10156_MOESM1_ESM.docx]

# Additional file 1

**Sub study: Qualitative Comparative Analysis (QCA)**

The present analysis was part of the AdAM project (German: Anwendung für ein digital gestütztes Arzneimitteltherapie- und Versorgungsmanagement, or “application of digitally supported drug-therapy and care management”), which was conducted between July 2017 and June 2021.

The CDSS-based AdAM intervention addressed the medication management of multimorbid patients with polypharmacy performed by physicians (general practitioners). The original study protocol was published in 2021:

*Müller, B. S., Klaaßen-Mielke, R., Gonzalez-Gonzalez, A. I., Grandt, D., Hammerschmidt, R., Köberlein-Neu, J., ... & Muth, C. (2021). Effectiveness of the application of an electronic medication management support system in patients with polypharmacy in general practice: a study protocol of cluster-randomised controlled trial (AdAM). BMJ open, 11(9), e048191.*
