# Supplementary material for: Factors facilitating the implementation of a clinical decision support system in primary care practices: a fuzzy set qualitative comparative analysis
Source: BMC Health Serv Res. 2023 Oct 26;23:1161. doi: 10.1186/s12913-023-10156-9 (PMC10605331; doi:10.1186/s12913-023-10156-9)
Supplement: Supplementary file 2 — Additional file 2. AdAM survey (translated version). [file 12913_2023_10156_MOESM2_ESM.docx]

Additional file 2

**AdAM survey (translated version)**

1. **Dealing with change in your practice team.**

Please indicate your level of agreement with the following statements regarding your practice team.

|  | strongly disagree | disagree | neither agree nor disagree | agree | strongly agree |
| --- | --- | --- | --- | --- | --- |
| We have the time resources to adequately dedicate ourselves to such a project | **□** | **□** | **□** | **□** | **□** |
| We have the human resources (education and skills of the employees) to adequately dedicate ourselves to such a project | **□** | **□** | **□** | **□** | **□** |

Please indicate your level of agreement with the following statements regarding your practice team.

| The *Practice Adaptive Reserve* measure (Jaén et al. 2010) | strongly disagree | disagree | neither agree nor disagree | agree | strongly agree |
| --- | --- | --- | --- | --- | --- |
| We regularly take time to consider ways to improve how we do things | **□** | **□** | **□** | **□** | **□** |
| People in our practice actively seek new ways to improve how we do things | **□** | **□** | **□** | **□** | **□** |
| People at all levels of this office openly talk about what is and isn’t working | **□** | **□** | **□** | **□** | **□** |
| People are aware of how their actions affect others in this practice | **□** | **□** | **□** | **□** | **□** |
| Most people in this practice are willing to change how they do things in response to feedback from others | **□** | **□** | **□** | **□** | **□** |
| This practice encourages everyone (front office staff, clinical staff, nurses, and clinicians) to share ideas | **□** | **□** | **□** | **□** | **□** |
| I can rely on the other people in this practice to do their jobs well | **□** | **□** | **□** | **□** | **□** |
| Difficult problems are solved through face-to-face discussions in this practice | **□** | **□** | **□** | **□** | **□** |
| We regularly take time to reflect on how we do things | **□** | **□** | **□** | **□** | **□** |
| After trying something new, we take time to think about how it worked | **□** | **□** | **□** | **□** | **□** |
| The practice leadership makes sure that we have the time and space necessary to discuss changes to improve care | **□** | **□** | **□** | **□** | **□** |
| Leadership in this practice creates an environment where things can be accomplished | **□** | **□** | **□** | **□** | **□** |
| Practice leadership promotes an environment that is an enjoyable place to work | **□** | **□** | **□** | **□** | **□** |
| Leadership strongly supports practice change efforts | **□** | **□** | **□** | **□** | **□** |
| This practice learns from its mistakes | **□** | **□** | **□** | **□** | **□** |
| It is hard to get things to change in our practice (reverse score) | **□** | **□** | **□** | **□** | **□** |
| Mistakes have led to positive changes here | **□** | **□** | **□** | **□** | **□** |
| People in this practice have the information that they need to do their jobs well | **□** | **□** | **□** | **□** | **□** |
| When we experience a problem in the practice, we make a serious effort to figure out what’s really going on | **□** | **□** | **□** | **□** | **□** |
| I have many opportunities to grow in my work | **□** | **□** | **□** | **□** | **□** |
| People in this practice operate as a real team | **□** | **□** | **□** | **□** | **□** |
| Most of the people who work in our practice seem to enjoy their work | **□** | **□** | **□** | **□** | **□** |
| This practice is a place of joy and hope | **□** | **□** | **□** | **□** | **□** |

Please indicate your level of agreement with the following statements regarding your practice team.

| The *Organizational readiness for implementing change* measure (Shea et al. 2014)^[[1]](#footnote-1)^ | strongly disagree | disagree | neither agree nor disagree | agree | strongly agree |
| --- | --- | --- | --- | --- | --- |
| People who work here are committed to implementing the AdAM intervention | **□** | **□** | **□** | **□** | **□** |
| People who work here will do whatever it takes to implement this change | **□** | **□** | **□** | **□** | **□** |
| People who work here want to implement the AdAM intervention | **□** | **□** | **□** | **□** | **□** |
| People who work here are determined to implement the AdAM intervention | **□** | **□** | **□** | **□** | **□** |
| People who work here are motivated to implement the AdAM intervention | **□** | **□** | **□** | **□** | **□** |
| People who work here feel confident that they can handle the challenges that might arise in implementing the AdAM intervention | **□** | **□** | **□** | **□** | **□** |
| People who work here feel confident that they can keep track of progress in implementing the AdAM intervention | **□** | **□** | **□** | **□** | **□** |
| People who work here feel confident that they can coordinate tasks so that implementation goes smoothly | **□** | **□** | **□** | **□** | **□** |
| People who work here feel confident that the organisation can support people as they adjust to the AdAM intervention | **□** | **□** | **□** | **□** | **□** |

1. **Résumé**

Please give us your final feedback on the AdAM project by indicating your level of agreement with the following statements.

|  | strongly disagree | disagree | neither agree nor disagree | agree | strongly agree |
| --- | --- | --- | --- | --- | --- |
| The AdAM software is an enhancement of our existing technological equipment. | **□** | **□** | **□** | **□** | **□** |
| My expectations regarding the use of the AdAM software have been fulfilled. | **□** | **□** | **□** | **□** | **□** |
| The AdAM software gives me confidence in my decisions and actions in the context of my patients’ drug therapy. | **□** | **□** | **□** | **□** | **□** |
| The communication about the project by the project management (KVWL and BARMER) motivated me to introduce AdAM into my primary care practice. | **□** | **□** | **□** | **□** | **□** |
| The attempts by the project management to contact me during the project motivated me to use AdAM. | **□** | **□** | **□** | **□** | **□** |
| I used the AdAM software with all enrolled patients whenever necessary from my perspective. | **□** | **□** | **□** | **□** | **□** |

1. **Information about yourself and your practice**

| What is your gender? | **□**  female | | | **□**  male | | **□**  Non-binary / other | | |
| --- | --- | --- | --- | --- | --- | --- | --- | --- |
| How old are you? | **□**  30 to 40 years | **□**  41 to 50 years | | | **□**  51to 60 years | | | **□**  Older than 60 years |
| Do you work in a…? | **□**  Single practice | | **□**  Joint practice | | | | **□**  Medical Care Center | |
| How many physicians are working in your practice besides you? | **__ __** physicians besides me | | | | | | | |
| How many more people work in this practice? | **__ __** medical assistants  **__ __** practice manager  **__ __ ___________**  **__ __ ___________** | | | | | | | |
| How many years have you been working in this practice? | **__ __** years | | | | | | | |
| Have you participated in the AdAM training? | **□**  Yes | | | | **□**  No | | | |
| How many employees work with the AdAM intervention in your practice? | **__ __** employees | | | | | | | |

1. In this document, we applied the original English version of the ORIC measure by Shea et al. (2014) DOI: 10.1186/1748-5908-9-7. In the German version of this questionnaire, we used the German translation by Lindig et al. (2020) DOI: 10.1136/bmjopen-2019-034380. The German version consists of 9 items. [↑](#footnote-ref-1)
