# Supplementary material for: Factors facilitating the implementation of a clinical decision support system in primary care practices: a fuzzy set qualitative comparative analysis
Source: BMC Health Serv Res. 2023 Oct 26;23:1161. doi: 10.1186/s12913-023-10156-9 (PMC10605331; doi:10.1186/s12913-023-10156-9)
Supplement: Supplementary file 3 — Additional file 3. Checklist based on STANDARDS OF GOOD PRACTICE IN QUALITATIVE COMPARATIVE ANALYSIS (QCA) AND FUZZY-SETS by Schneider & Wagemann 2007 (doi:10.1163/156913210X12493538729793). [file 12913_2023_10156_MOESM3_ESM.docx]

Additional file 3

**Checklist based on STANDARDS OF GOOD PRACTICE IN QUALITATIVE COMPARATIVE ANALYSIS (QCA) AND FUZZY-SETS by Schneider & Wagemann 2007** (doi:10.1163/156913210X12493538729793)

| **Item** | **Recommendation** | **Text passage** |
| --- | --- | --- |
| **Criteria Concerning the Purpose of QCA** | | |
| a | QCA as a data analysis technique should be used for its original aims. | p. 5-6; 10 |
| b | QCA should not be applied as the only data analysis technique in a research project. | p. 8 |
| **Criteria Concerning the Research Strategy** | | |
| c | QCA should never be applied in a mechanical way; instead, it should always be related to the cases. | p. 10; 14-16 |
| d | Familiarity with the cases is a requirement before, during and after the analytical moment of a QCA analysis. | p. 10; 14-18 |
| **Criteria Concerning the Representation of QCA** | | |
| e | Whenever possible, the raw data matrix should be published. | N/A |
| f | The truth table should always be reported. | p. 13 |
| g | Every QCA analysis must contain the solution formula(s). | p. 14 |
| h | The consistency and coverage measures should always be reported. | p. 14 |
| i | The appropriate QCA terminology should be followed. | p. 10 ff. |
| j | As many forms of representing QCA results as needed should be used in order to depict both the case-oriented and the variable-oriented aspects of QCA. | p. 13 ff. |
| **Criteria for the Selection of Cases, Conditions, Set Memberships, and Truth Table Algorithm Criteria.** | | |
| k | There should always be an explicit and detailed justification for the (non)selection of cases | p. 10 |
| l | The conditions and the outcome should be selected and conceptualized on the basis of adequate theoretical and empirical prior knowledge | p. 11 ff. |
| m | The number of conditions should be kept moderate. | p. 11-12 |
| n | The dichotomization (csQCA) or calibration (fsQCA) of membership values should be discussed in detail. | p. 10/12 |
| o | In csQCA, contradictory truth table rows should be resolved before the minimization of the truth table algorithm | N/A (no contradictory rows occured, p.13) |
| **Criteria for the ‘Analytic Moment’s** | | |
| p | Use computer software to minimize the truth table. | p. 10 |
| q | Necessary and sufficient conditions should be analyzed in separate analytical steps. | Appendix E; p. 13-14 |
| r | The analysis of sufficient conditions should always be performed with and without simplifying assumptions regarding the logical remainders. Both solution formulas should be reported. | N/A  No logical remainders occured (see p.13) |
| s | The treatment of logical remainders should be transparent. | N/A  No logical remainders occured (see p. 13) |
| t | The treatment of contradictory rows (in csQCA) and of inconsistent truth table rows (in fsQCA) in the logical minimization process should be transparent. | N/A (no contradictory rows occured, see p. 13) |
| u | The outcome and the negation of the outcome should always be analyzed in two separate analyses | Outcome analysis: p.13-14;  Analysis of negated outcome: N/A (did not apply to the research question) |
| **Criteria for the Interpretation of Analytic Results** | | |
| v | Single conditions of a conjunctural and equifinal solution term should not be (over)interpreted. | p. 16 ff. |
| w | The researcher should always give explicit justifications in case one (or more) of the paths towards the outcome are deemed more important than others. | N/A |
| x | The solution formulas should be linked back to the cases. | p. 16 ff. |
| y | The coefficients of consistency and coverage are important components of the analysis and interpretation of QCA results. | Appendix F |
| z | The solution formula alone does not demonstrate a causal relationship between the conditions and the outcome. | p. 16 ff. |
