# Supplementary material for: Factors facilitating the implementation of a clinical decision support system in primary care practices: a fuzzy set qualitative comparative analysis
Source: BMC Health Serv Res. 2023 Oct 26;23:1161. doi: 10.1186/s12913-023-10156-9 (PMC10605331; doi:10.1186/s12913-023-10156-9)
Supplement: Supplementary file 4 — Additional file 4. STROBE Statement—Checklist of items that should be included in reports of observational studies. [file 12913_2023_10156_MOESM4_ESM.doc]

Additional file 4

**STROBE Statement—Checklist of items that should be included in reports of *observational studies.***

|  | Item No | Recommendation |
| --- | --- | --- |
| **Title and abstract** | 1 | (*a*) Indicate the study’s design with a commonly used term in the title or the abstract |
|  |  | (*b*) Provide in the abstract an informative and balanced summary of what was done and what was found |
| Introduction | | |
| Background/rationale | 2 | Explain the scientific background and rationale for the investigation being reported (p. 5-6) |
| Objectives | 3 | State specific objectives, including any prespecified hypotheses (p.6) |
| Methods | | |
| Study design | 4 | Present key elements of study design early in the paper (p.6, 8, 10) |
| Setting | 5 | Describe the setting, locations, and relevant dates, including periods of recruitment, exposure, follow-up, and data collection (p.8-9) |
| Participants | 6 | (*a*) Give the eligibility criteria, and the sources and methods of selection of participants (p.9, 12) |
| Variables | 7 | Clearly define all outcomes, exposures, predictors, potential confounders, and effect modifiers. Give diagnostic criteria, if applicable (p.11-12) |
| Data sources/ measurement | 8* | For each variable of interest, give sources of data and details of methods of assessment (measurement). Describe comparability of assessment methods if there is more than one group (Table 2) |
| Bias | 9 | Describe any efforts to address potential sources of bias (p.9) |
| Study size | 10 | Explain how the study size was arrived at (p.8-9) |
| Quantitative variables | 11 | Explain how quantitative variables were handled in the analyses. If applicable, describe which groupings were chosen and why (p.11-12) |
| Statistical methods | 12 | (*a*) Describe all statistical methods, including those used to control for confounding (p.10-12) |
|  |  | (*b*) Describe any methods used to examine subgroups and interactions (p.10-11) |
|  |  | (*c*) Explain how missing data were addressed (p.9) |
|  |  | (*d*) If applicable, describe analytical methods taking account of sampling strategy (N/A) |
|  |  | (*e*) Describe any sensitivity analyses (Appendix F) |
| Results | | |
| Participants | 13* | (a) Report numbers of individuals at each stage of study—eg numbers potentially eligible, examined for eligibility, confirmed eligible, included in the study, completing follow-up, and analysed (Table 1; p.9) |
|  |  | (b) Give reasons for non-participation at each stage (N/A) |
|  |  | (c) Consider use of a flow diagram (N/A) |
| Descriptive data | 14* | (a) Give characteristics of study participants (eg demographic, clinical, social) and information on exposures and potential confounders (Table 1) |
|  |  | (b) Indicate number of participants with missing data for each variable of interest (N/A) |
| Outcome data | 15* | Report numbers of outcome events or summary measures (p.13-14) |
| Main results | 16 | (*a*) Give unadjusted estimates and, if applicable, confounder-adjusted estimates and their precision (eg, 95% confidence interval). Make clear which confounders were adjusted for and why they were included (N/A) |
|  |  | (*b*) Report category boundaries when continuous variables were categorized (N/A) |
|  |  | (*c*) If relevant, consider translating estimates of relative risk into absolute risk for a meaningful time period (N/A) |
| Other analyses | 17 | Report other analyses done—eg analyses of subgroups and interactions, and sensitivity analyses (p.14-15) |
| Discussion | | |
| Key results | 18 | Summarise key results with reference to study objectives (p.13-14) |
| Limitations | 19 | Discuss limitations of the study, taking into account sources of potential bias or imprecision. Discuss both direction and magnitude of any potential bias (p.23) |
| Interpretation | 20 | Give a cautious overall interpretation of results considering objectives, limitations, multiplicity of analyses, results from similar studies, and other relevant evidence (p.16f.) |
| Generalisability | 21 | Discuss the generalisability (external validity) of the study results (p.2-21) |
| Other information | | |
| Funding | 22 | Give the source of funding and the role of the funders for the present study and, if applicable, for the original study on which the present article is based (p.28) |

*Give information separately for exposed and unexposed groups.
