# Supplementary material for: Factors facilitating the implementation of a clinical decision support system in primary care practices: a fuzzy set qualitative comparative analysis
Source: BMC Health Serv Res. 2023 Oct 26;23:1161. doi: 10.1186/s12913-023-10156-9 (PMC10605331; doi:10.1186/s12913-023-10156-9)
Supplement: Supplementary file 5 — Additional file 5. Necessity analysis. [file 12913_2023_10156_MOESM5_ESM.docx]

# Additional file 5

## Necessity analysis

Before analyzing the truth table, we checked whether any condition is by itself necessary for the presence of the outcome. Table A1 shows the necessity analysis for all conditions and their negations. None meet the consistency threshold of .90 (1). Thus, no condition is by itself necessary for implementation success.

Table A 1 Results of necessity analysis.

| **Condition** | **Inclusion** | **Coverage** |
| --- | --- | --- |
| *Strong Inside Motivation* | .676 | .744 |
| ~*Strong Inside Motivation* | .607 | .535 |
| *High Capability for Development* | .611 | .743 |
| ~*High Capability for Development* | .684 | .560 |
| *Strong Outside Motivation* | .606 | .772 |
| ~*Strong Outside Motivation* | .675 | .536 |
| *Many Options for Development* | .633 | .790 |
| ~*Many Options for Development* | .651 | .524 |

*Note*: Tilde (~) indicates the negation of the respective condition.

Literature Cited

1. Greckhamer T, Furnari S, Fiss PC, Aguilera RV. Studying configurations with qualitative comparative analysis: Best practices in strategy and organization research. Strategic Organization 2018; 16(4):482–95.
