# Supplementary material for: Factors facilitating the implementation of a clinical decision support system in primary care practices: a fuzzy set qualitative comparative analysis
Source: BMC Health Serv Res. 2023 Oct 26;23:1161. doi: 10.1186/s12913-023-10156-9 (PMC10605331; doi:10.1186/s12913-023-10156-9)
Supplement: Supplementary file 6 — Additional file 6. Robustness test. [file 12913_2023_10156_MOESM6_ESM.docx]

# **Additional file 6**

## Robustness test

We tested the robustness of our results according to the Robustness Test Protocol by Oana and Schneider (1) using the R package SetMethods (1). The protocol comprises three steps covering the following properties:

Sensitivity ranges, Fit-oriented robustness and Case-oriented robustness.

**1. Sensitivity ranges**

In the first step, we identified the sensitivity ranges of our consistency and frequency thresholds. Stepwise, we changed one threshold while holding everything else constant to determine the limits within which our solution remains the same. Table B1 shows that incremental changes in both consistency and frequency thresholds affect our results. To assess the impact of these changes on our results, we proceed with the next step.

**2. Fit-oriented robustness**

Knowing that our initial solution (IS) is susceptible to changes, we tested the robustness of our IS against other logically possible solutions (test solutions [TSs]):

1. TS1: We increased the frequency threshold from 1 to 2.
2. TS2: We increased the consistency threshold from .8 to .81.
3. TS3-6: We lowered the exclusion cutoff for the calibration of each condition from 2 to 1.9. Thus we judged cases to be fully out of the respective sets only if they rated (on average) less than “*disagree*”. Since we adjusted each of the four conditions separately, we tested four additional test solutions, TSs 3-6: Each included changes in solely the Strong Inside Motivators (TS3), the High Capability for Development (TS4), the Strong Outside Motivators (TS5) and the Many Options for Development (TS6).

The effect of these changes on our IS are only minor (see Table B2). Accordingly, the fit-oriented robustness parameters close to 1 (see Table B1) indicate that our IS is robust (see RF_cov_ and RF_cons_) and that it largely overlaps with our TSs (TS1-6) (see RF_SCmin_ and RF_SCmax_).

**3. Case-oriented robustness**

In the third and final step, we assessed the robustness of our individual cases. The XY plot (Figure B1) depicts case memberships in the IS (X-axis) and in the minimum of all TSs (Y-axis). While most of our cases are exactly on the diagonal, meaning they are part of both the IS and the minimum of our TSs, our results show a few shaky cases (lower right quadrant). Shaky cases are part of the IS but not part of any TS. Consequently, our results yielded a robustness rank of 3 on a scale from 1 (no shaky or possible cases) to 4 (both shaky and possible cases). However, the case-oriented robustness parameters (RCR_typ_ and RCR_dev_) close to 1 indicate that these shaky cases only account for a low share of cases in our IS.

We conclude that our IS is sensitive to the modification of several thresholds. However, these modifications only cause minor changes in our IS, as indicated by the fitness-oriented robustness parameters close to 1. At the individual-case level, we find a few shaky cases. However, since we conducted a large-*n* study, these shaky cases only account for a low share of overall cases included in our IS. Thus, we find our IS to be sufficiently robust and continue with the interpretation of our results.

Literature Cited

1. Oana I-E, Schneider CQ. A Robustness Test Protocol for Applied QCA: Theory and R Software Application. Sociological Methods & Research 2021:004912412110361.

Table B1 Robustness Protocol Report.

| **Sensitivity Ranges** | | | | |
| --- | --- | --- | --- | --- |
| **Parameters** | Raw Consistency | Lower: .77 | Upper: .8 |  |
|  | Frequency | Upper: 1 | Upper: 2 |  |
| **Robustness Parameters** | | | | |
| **Fit Oriented** | RF_cons_: .98 | RF_cov_: .88 | RF_SC_minTS_: .86 | RF_SC_maxTS_: .98 |
| **Case Oriented** | RCR_typ_: .85 | RCR_dev_:.88 | RCC_Rank: 3 |  |

Table B2 Comparing parsimonious solutions for initial solution and test sets

|  | **Solution** | | **TS1** | | **TS2** | | **TS3** | | **TS4** | | **TS5** | | **TS6** | |
| --- | --- | --- | --- | --- | --- | --- | --- | --- | --- | --- | --- | --- | --- | --- |
|  | **1** | **2** | **1** | **2** | **1** | **2** | **1** | **2** | **1** | **2** | **1** | **2** | **1** | **2** |
| Strong Inside Motivators |  | 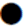 | 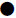 | 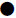 | 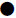 | 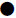 | 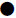 | 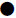 | 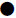 | 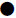 | 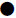 | 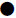 | 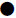 | 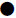 |
| High Capability for Development | 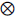 | 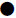 | 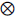 | 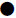 | 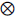 | 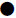 | 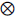 | 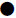 |  | 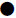 | 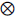 | 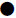 |  | 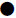 |
| Strong Outside Motivators |  | 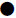 |  | 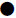 |  |  |  |  | 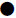 | 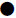 |  | 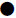 | 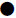 | 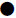 |
| Many Options for Development | 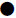 |  | 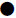 |  | 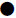 | 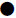 | 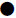 | 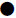 | 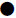 |  | 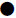 |  | 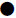 |  |
| Consistency | .86 | .87 | .86 | .87 | .86 | .87 | .86 | 87 | .88 | .87 | .86 | .86 | .87 | .87 |
| Raw Coverage | .4 | .44 | .4 | .44 | .4 | .44 | .4 | .45 | .45 | .45 | .4 | .45 | .45 | .44 |
| Unique Coverage | .1 | .15 | .1 | .15 | .1 | .15 | .1 | .15 | .05 | .04 | .1 | .15 | .04 | .04 |
| **Overall Solution Consistency** | **.85** | | **.85** | | **.85** | | **.85** | | **.86** | | **.85** | | **.87** | |
| **Overall Solution Coverage** | **.54** | | **.54** | | **.54** | | **.55** | | **.49** | | **.55** | | **.49** | |


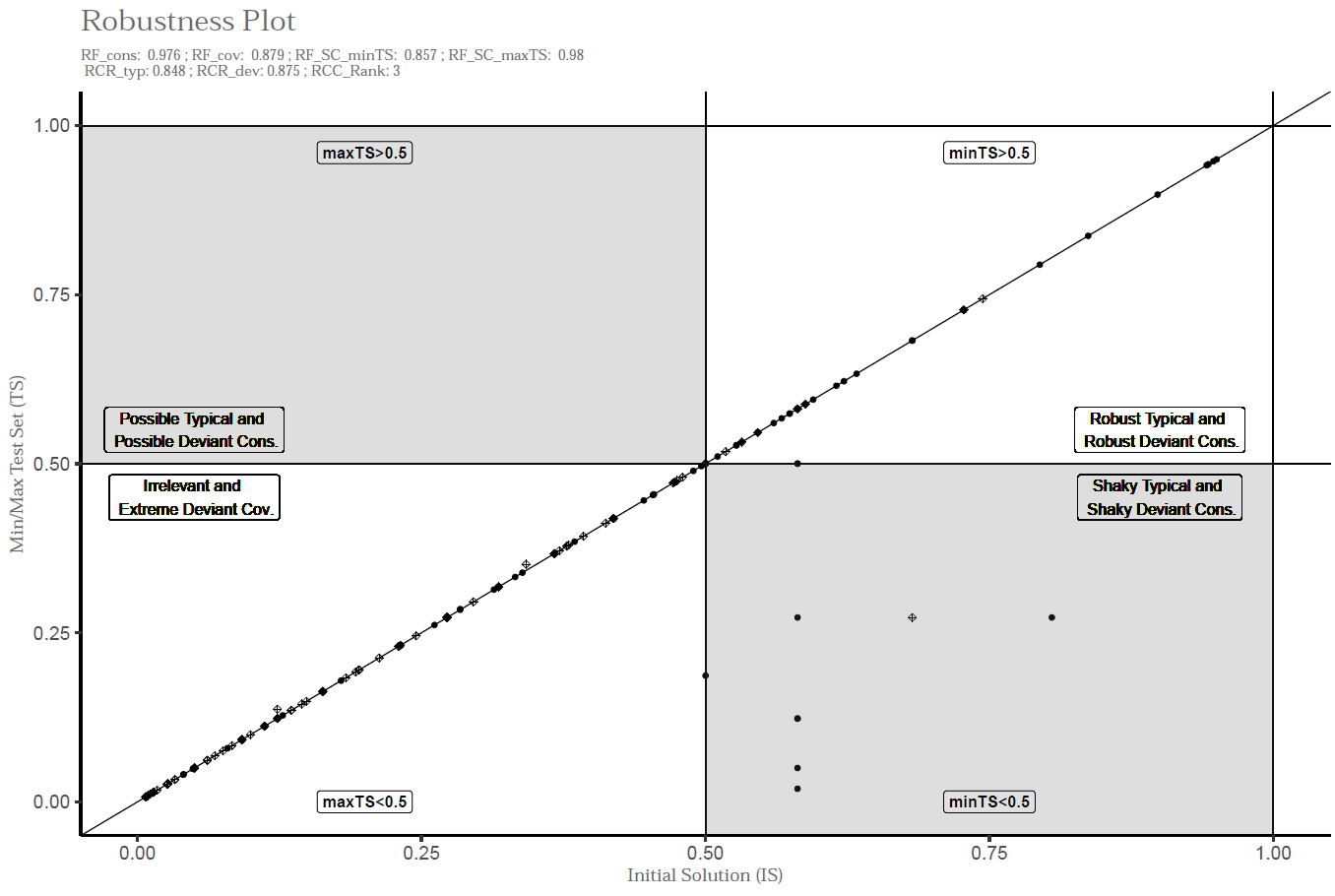


Figure B1 Robustness Test, XY Plot.
